# Leadership in healthcare during a pandemic: for a systems leadership approach

**DOI:** 10.3389/fpubh.2024.1361046

**Published:** 2024-05-22

**Authors:** Camille Castelyn

**Affiliations:** ^1^Albert Luthuli Leadership Institute, Faculty of Economic and Management Sciences, University of Pretoria, Pretoria, South Africa; ^2^Centre for Ethics and Philosophy of Health Sciences, Faculty of Health Sciences, University of Pretoria, Pretoria, South Africa

**Keywords:** leadership, social determinants of health, pandemic, systems approach, public health

## 1 Introduction

During the COVID-19 pandemic, it became clear that the need for leadership and leadership development is more important than ever. The leadership and policy failures during the COVID-19 pandemic are estimated at $125–200 billion in incremental costs to annual health care expenditures in United States alone (1). There have been several pandemics in the past 20 years and it is essential that these leadership failures are addressed for future pandemics (2). On September 19, 2023, the United Nations unanimously adopted the Political Declaration on Pandemic Prevention, Preparedness, and Response (PPPR), affirming that pandemics call for timely, urgent, and continued leadership (3). According to the World Health Organization, leadership and governance are two of the six building blocks of health systems. The other building blocks are service delivery, health workforce, health information, medical technologies, and health financing (4).

The concept of leadership has been under scrutiny for many years, moving from a leader-centric to a people-oriented approach. Crisis leadership is one such a leader-centric approach. Crisis leadership in various pandemic situations, as per Sriharan et al.'s review, entails performing tasks such as preparation, planning, communication and collaboration (1). Healthcare leaders are responsible for identifying the crisis early, developing emergency preparedness protocols, monitoring crises, implementing protocols, allocating resources, and developing contingency plans (1). They are expected to provide empathy and awareness, inspire and influence decision-making, provide systems thinking and sense-making, develop tacit skills, and build political collaboration (1). Although these are sensible traits to develop in healthcare leaders, leadership studies have moved beyond simply a laundry list of amiable traits toward a systems thinking approach.

### 1.1 Discussion: a systems leadership and critical theory approach

Public healthcare systems require a systems leadership approach (5, 6). A systems leadership approach entails acknowledging that healthcare systems are complex (5, 6). The complexity of healthcare systems lies in the constant interplay between people, culture, economics, and the healthcare infrastructure. People are central to healthcare systems and are thus the drivers of the system (4). Their agency, mindsets and power discourses are thus driving factors within this system (5). A systems leadership approach differs from older concepts of leadership in that it goes beyond organizational and professional boundaries in order to address “wicked problems.” (7). It is a collective approach, meaning many people working at different levels and in different places, crossing boundaries (7).

A systems leadership approach is especially practical within the African context. This was echoed at the 3rd International Conference on Public Health in Africa that took place at Mulungushi International Conference Center, Lusaka, Zambia, from November 27–30, 2023. Ambassador Lewis Brown, a former Minister of Information, Cultural Affairs, and Tourism in Liberia, delivered his keynote speech, “*Transformative Leadership for Health in Africa”*, emphasizing that Africa simply cannot, should not, and will not let others do what they must do for themselves, they must seek solutions for Africans by Africans and develop a continental strategy. Constructing public health institutions to address Africa's leadership in health diplomacy should be prioritized. It was acknowledged that the size of the public healthcare challenge in Africa is not to be underestimated. For example, 94% of cases of malaria worldwide are in the WHO African region. (8) Another 12.7 million children in Africa missed out on one or more vaccinations in the past 3 years (9). Furthermore, it is expected that in Southern Africa people over the age of 60 will triple between 2020 and 2050. Ambassador Brown emphasized the importance of maximizing technologies, scaling up, investing in human capital and capacity building, supporting community healthcare workers, preparing for the changing landscape of climate migration, and using evidence-based approaches. He asked the audience to imagine what the future of health would look like and who would be waving the public health flag in the future. He emphasized that public health systems should not wait for patients to come to them but that they must go to them. He said that one of the biggest priorities should be shaping political reforms that are able to prioritize the equitable allocation of resources. He urged people to not have a negative connotation for politics, stating that public health must become an inclusive culture and not a disconnected refuge for scientists. People should feel taken care of from the cradle to the grave. To accomplish all of this, he said that it is of utmost importance to strengthen without compromising African agency as well as leadership and accountability. He envisions a new public health order whereby leadership will mean renewing Africa's position as secure in healthcare politics and encouraging other global bodies to do the same. He ended by stating that the time for this is now, as many lives, including all the lives in this room, depend on it.

This definition of leadership aptly sums up a systems leadership approach:

“Leadership is a social process of influence – there are things people can do to enhance specific skills and their ability to cope with situations but the processes and outcomes of leadership remain socially embedded – the result of a complex interaction between a multitude of factors. Thus who becomes a leader how they behave, and what they do are all determined by social and cultural factors as by any individual characteristics – Church, Hitler, Stalin, Gandhi and King were all products of their time, place and culture” (10).

Cultural factors, political factors, and structural factors play a role in shaping leadership competencies; however, due to the complexity of health systems, no causal relationships are established (1). For future pandemic preparedness, cultural, political, and structural factors need to be accounted for. According to Sriharan et al., cultural factors have to do with the way in which people communicate, collaborate, build relationships, and make decisions (1). They found that improved trust between leaders and stakeholders improved collaboration. Improved collaboration can be achieved through transparent communication (1). Gender roles also form part of the cultural context of leadership, which is ultimately formed by social norms and plays an important role too. Political factors include power dynamics among various levels of governance and the leader's ability to influence resource allocation (1). Distrust was also featured as a major stumbling block by means of public trust in communications by public health care leaders (1). Structural factors such as lack of team cohesiveness' and centralized control delaying decision-making were also influential (1). Cultural, political, and structural factors should thus be addressed by better training to prepare the workforce (1). An example of this is the Albert Luthuli Leadership Platform for Health, situated at the University of Pretoria, which aims to reimagine health leadership by offering courses in global health leadership.

Ultimately, healthcare systems are an intersection between systems and the lifeworlds of people living within these systems. The theory of lifeworld and systems, can be attributed to Habermas' communicative action and discourse ethics theories, which stemmed from the development of critical theory. Critical theory emerged during the enlightenment period. It is a socio-philosophical school of thought. Critical theory aims to “analyze social conditions, to criticize the unjustified use of power, and to change established social traditions and institutions so that human beings are freed from dependency, subordination, and suppression. Critical theory is oriented toward the development of a more humane, rational, and just society” (11). Habermas in the 1950s contributed significantly to this approach. In his Theory of Communicative Action, he refers to the work of Parsons, Weber, and Schutz, distinguishing between lifeworld and system (11). Lifeworld is the social world that is based on the taken-for-granted social skills and knowledge of members within the lifeworld (11). It is constructed and maintained through interactions and conversations between ordinary people, thereby building on communicative reason in order to establish a shared understanding of the world as a meaningful place (11). Whereas, system, is the result of differentiation and specialization in modern society (11).

### 1.2 Discussion: example of a systems leadership thinking approach in the South African public healthcare context

During the COVID-19 pandemic in South Africa, forensic pathologists reportedly saw many forgotten populations suffering in the form of being isolated and unable to access necessary medical care. In a global aging population, South Africa has the fastest-growing older adult community, where 8.1% of population is more than 60 years old (12). The Older Persons Act 13 was adopted in 2006 and aims to deal effectively with the plight of older persons by establishing a framework aimed at the empowerment and protection of older persons and at the promotion and maintenance of their status, rights, wellbeing, safety, and security. In terms of family law, an older person is the person who, in the case of a male, is 65 years of age or older and, in the case of a female, is 60 years of age or older. An abuse of an older person occurs when any person, in a relationship where there is an expectation of trust, does something or fails to do something that causes harm or distress or is likely to cause harm or distress to an older person.

In 2021, President Cyril Ramaphosa was appointed by the African Union Bureau of Assembly of Heads of States and Government as its champion on COVID-19, establishing the African COVID-19 Response (13). His approaches included developing an endorsed continental strategy for the COVID-19 outbreak, establishing the Africa Task Force for Coronavirus, developing and establishing the African Medical Supplies Platform, establishing the COVID-19 African Vaccine Acquisition Task Team to secure financing for and acquire vaccines, coordinating communications and contributions across the continent on COVID-19 matters (13).

There are mixed responses regarding whether Cyril Ramaphosa's leadership approach was good or bad; this can be based on biases and perceptions. Leadership scholarship studies differentiate between leadership development and unpacking the phenomenon of leadership. In a world where we easily call out a good or bad leader, critical leadership scholarly studies are necessary to help guide our perceptions and evaluations. This is especially important in these times. The social determinants of health and a global aging population have indicated that the world we live in is increasingly complex. We therefore need understanding and approaches that consider this complexity without complicating things.

## 2 Practical steps toward a systems leadership approach

Six practical points have been identified by Bigland et al. that may aid South Africa and other countries in future systems leadership approaches (6). First is a call to develop a call to action for gathering a “coalition of the willing”. A coalition of the willing includes a wide variety of people, including the actual population. Second, there is a need for a dedicated system coordinator role; this enables efficient decision-making. Third, relationship building by incorporating trust and sharing vulnerabilities should be incorporated. Fourth, building resilient systems by being flexible and adapting behaviors to the context needs to be prioritized. Fifth, being able to hold space for paradoxes around power, uncertainty and conflict within the systems is critical. These paradoxes should not be seen as something to overcome but as something to be worked with constructively and dynamically to drive meaningful action and progress. This means that issues need to be recognized and that leaders need to be trained to work with confidence in spaces of uncertainty. Lastly, the ways in which the effectiveness of systems is measured should be widened to include a wider diversity of experiences. For example, a sense of pleasure and shared endeavor among colleagues should be highly valued.

Pandemic preparedness will surely remain essential in the coming years, and a systems leadership approach may prove invaluable in ensuring that the leadership failures of the past are not repeated. A “coalition of willing”, people who are willing to work toward more just and equitable systems are what may make the difference, as we aim to fly the public health flag for all into a future where more people are able to flourish and lead meaningful lives. Questioning the dominant discourses and power structures within these systems is also essential if we are to truly build resilient systems.

## Author contributions

CC: Conceptualization, Data curation, Formal analysis, Funding acquisition, Investigation, Methodology, Project administration, Resources, Software, Supervision, Validation, Visualization, Writing – original draft, Writing – review & editing.
